# Renewed Attention Needed for Prevention of Sudden Unexpected Death in Infancy in the Netherlands

**DOI:** 10.3389/fped.2021.757530

**Published:** 2021-12-06

**Authors:** Floortje Kanits, Monique P. L'Hoir, Magda M. Boere-Boonekamp, Adèle C. Engelberts, Edith J. M. Feskens

**Affiliations:** ^1^Department of Human Nutrition and Health, Wageningen University, Wageningen, Netherlands; ^2^Community Health Centre, GGD Noord-Oost-Gelderland, Warnsveld, Netherlands; ^3^Department of Health Technology and Services Research, University of Twente, Enschede, Netherlands; ^4^Department of Pediatrics, Zuyderland Medical Centre, Sittard-Geleen, Netherlands

**Keywords:** SUDI (sudden unexpected death in infancy), SIDS (sudden infant death syndrome), prevention, safe sleeping, advice, parental behavior, surveys

## Abstract

**Background:** The incidence of sudden unexpected death in infancy (SUDI), which includes sudden infant death syndrome (SIDS), has declined in developed countries since the 1980s, including the Netherlands. To identify improvement opportunities in SUDI prevention, we monitored the adherence of parents to the prevention advice on infant care habits over the past 20 years, especially in relation to the SUDI incidence over time. Potential changes in parental adherence between the latest surveys are of specific interest, as these indicate where current focus is needed.

**Methods:** Description of the prevalence of infant care factors related to the risk of SUDI, assessed from five Dutch national surveys from 1999 to 2017 among parents of infants under 12 months, and analysis of the potential differences in these prevalences between the two latest surveys in 2010/11 and 2017 with a z-test.

**Results:** Supine sleeping position decreased from the highest prevalence of 92% in 2010/11, to 83% in 2017. Sleep sack use has increased to 55%, the highest prevalence up to now. Avoiding a duvet has remained reasonably stable since 2002/03 and now 95% of parents do not use a duvet. The prevalence of room-sharing, without sharing the bed, increased from 14% in 1999 to the highest prevalence in 2017 (31%). However, also bed-sharing almost doubled from 5.6% in 2010/11 to 10% in 2017. Breastfeeding decreased between 1999 and 2010/11, but increased from 34% in 2010/11 to 42% in 2017. An increased prevalence of mothers who abstained from smoking during pregnancy, as well as both parents not smoking, was observed, although mostly higher educated parents showed this beneficial behavior.

**Discussion and Conclusion:** Much has already been achieved first by decreasing prone sleeping since the 80's, and subsequently promoting supine as the safest sleep position. The decrease in duvet use and smoking, and an increase in breastfeeding have also had impact. Indications of a recent decreased prevalence of the supine sleeping position and higher prevalence of bed-sharing might relate to the slightly increasing SUDI incidence in the Netherlands. Renewed attention for prevention of SUDI and specific advice targeting high-risk groups is needed. Modern, picture driven information *via* internet is recommended.

## Introduction

**Figure 1:**
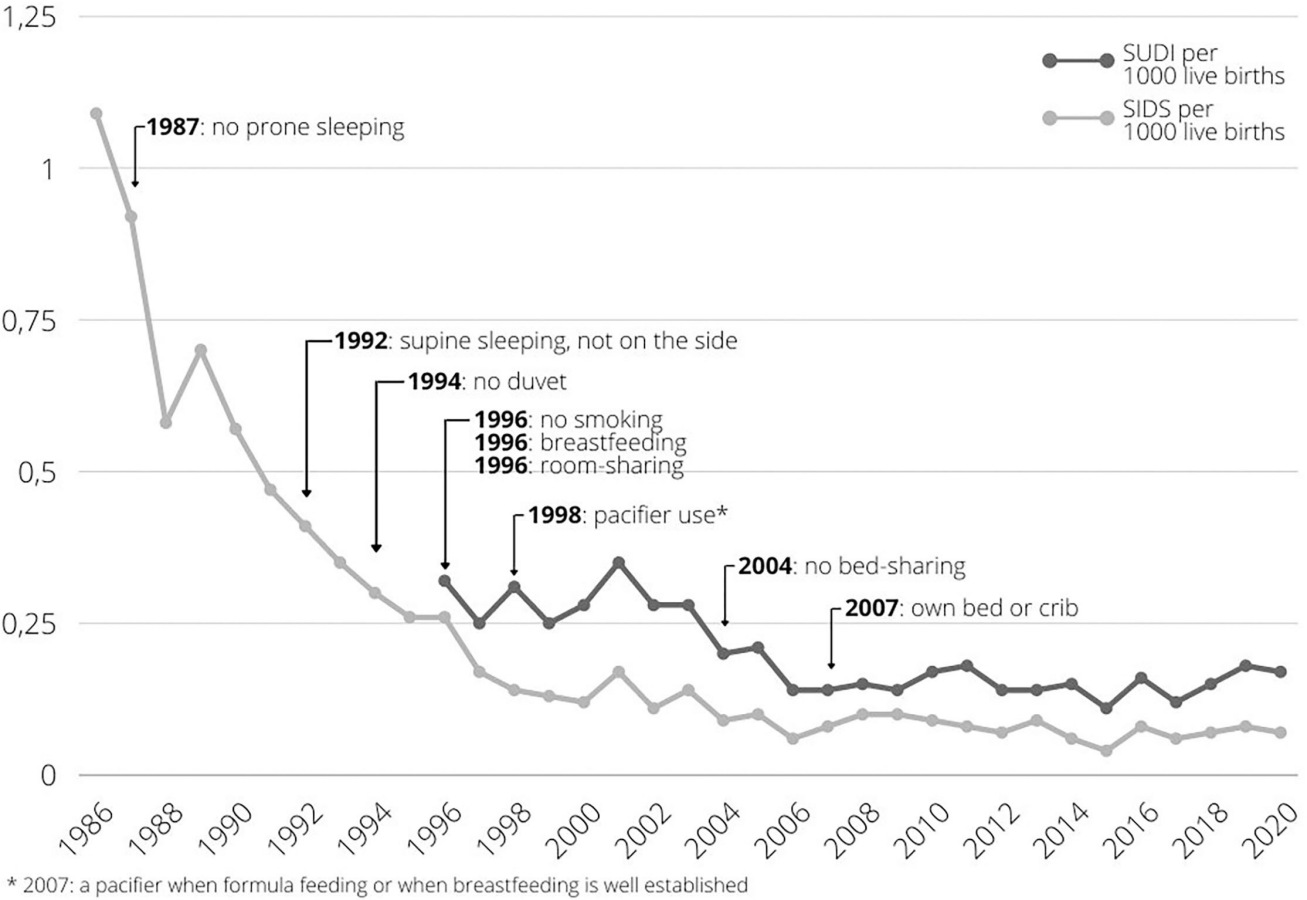
SUDI and SIDS incidence in the Netherlands from 1986 to 2019, with addition of prevention advice elements over time. Data retrieved from Central Bureau for Statistics Netherlands (4). SIDS incidence represents cases coded as R95 in the International Statistical Classification of Diseases and Related Health Problems, 10th Revision (ICD-10). SUDI incidence represents cases coded as R95, and R96: other sudden death, cause unknown, R98 unattended death, R99: other ill-defined and unspecified causes of mortality, W75: accidental suffocation and strangulation in bed, W78: inhalation of gastric contents, W79: inhalation and ingestion of food, causing obstruction of respiratory tract in the ICD-10, as suggested by Taylor et al. (3).

Sudden infant death syndrome (SIDS), also known as cot death, is defined as “the sudden unexpected death of an apparently healthy infant under 1 year of age that remains unexplained after a thorough case investigation, including performance of a complete autopsy with ancillary testing, examination of the death scene, and review of the clinical history” as suggested at the 3rd International Congress on Unexplained Deaths in Infants and Children (1, 2). Because of the differences in diagnosis and classification between countries, scientists now advocate the use of the term Sudden Unexpected Death in Infancy (SUDI), which includes SIDS (3). The comprehensive set of diagnostic categories used to define SUDI are specified in the footnote of Figure 1.

Both the incidence of SIDS and SUDI have declined in developed countries since the 1980s, as in the Netherlands, when the advice was given to place infants to sleep in the supine position (5, 6). Between 2002 and 2010, low incidence rates were found in the Netherlands (0.19 per 1,000 live births) (3). In the Netherlands, the SUDI rate has followed a trend similar to the SIDS rate over the years, and is slightly increasing again since 2017 (Figure 1). In 2019, 31 infants died suddenly and unexpectedly, of which 13 were classified as SIDS (4). The death of a seemingly healthy infant without an apparent cause, and without any warning, has an immense impact on parents, family and friends, causing them great grief (7).

The decline in SIDS and SUDI incidence in the Netherlands is attributable to ongoing prevention based on knowledge of risk factors (8), and the prevalence of these factors in the population. From 1987 onwards, 11 safe sleeping surveys were distributed in the Netherlands with time intervals varying from 1 to 7 years (9–18). Monitoring infant care practices by repeated national surveys is part of the SUDI prevention and provides input for preventive messages, including adjustments to existing messages.

In Figure 1, addition of SUDI prevention advice over time is visualized. This led to the latest Dutch guidelines for the prevention of SIDS and SUDI including the advice to always place an infant to sleep in the supine position, to make sure the infant is not too warm when in bed, not to smoke during and after pregnancy, to place the infant to sleep in a safe sleeping environment and to provide a safe situation when the infant is awake (8). This includes placing an infant to sleep in an own bed or crib in the parents' bedroom the first 6 months, not to bed-shareat least up to 4 months of age, and up to 6 months when one of the parents smokes. Additionally, breastfeeding is recommended. Based on this guideline, the Dutch Consumer Safety Institute (VeiligheidNL) currently promotes four major safe sleeping messages: place the infant to sleep on its back, in its own bed or cot, in an empty bed or cot, and in a well-fitting sleep sack (19). These advices are communicated to parents *via* midwives, maternity caregivers and child healthcare centers.

Especially since the incidence of SUDI is low, attention for prevention advice can weaken for both parents and (professional) caregivers, as well as governmental organizations and (public) health professionals, with increasing incidence rates as a result. Furthermore, popular infant care trends like sharing a sleep surface with an infant, shared on the internet and shown in magazines, may also influence the behavior of parents. A major part of the images online and in magazines do not adhere to the infant safe sleeping guidelines (20, 21). When the prevalence of behavior contrary to safe sleeping advice increases over time, a rise in SUDI incidence might also be expected. To identify improvement opportunities in SUDI prevention, it is important to monitor the adherence of parents to the advice, especially in relation to the SUDI incidence over time. Therefore, the aim of this study was to describe the prevalence of parental behavior recommended in the national infant safe sleeping guidelines. Potential changes in parental adherence between the latest surveys (2010/11 and 2017) are of specific interest, as these indicate where current focus in prevention is needed.

This resulted in the following research question: How can the development of infant care behavior of parents over the past 20 years in the Netherlands be described, and what are the differences in the prevalence of these behaviors between 2010/11 and 2017?

## Materials and Methods

### Design

This descriptive study based on successive independent samples including five latest cross-sectional studies, describes the prevalence of infant care factors related to the risk of SUDI, assessed from Dutch national surveys from 1999 to 2017, and analyses the differences in prevalence between the two latest surveys in 2010/11 and 2017.

### Data Collection

Data of five Dutch safe sleeping surveys were used, each of them representing sleeping conditions prevalences of a sample of the general population. These surveys were consecutively conducted by the Dutch organization for applied scientific research (TNO) in 1999 (*N* = 2,534), 2002/03 (*N* = 2,869) and 2010/11 (*N* = 1,956), the National Cot Death Working group (LWW) of the Dutch Pediatric Society (NVK) in 2005 (*N* = 1,399) and the University of Twente in 2017 (*N* = 1,209). Original data of these surveys were used, except for 1999, where only published results were available.

Survey administration differed slightly between the five successive surveys. For all surveys, Child Health Care (CHC) organizations in the Netherlands were asked to contribute by selecting all, or a random sample of CHC centers in their working area. Details of data collection are described in Table 1.

**Table 1:** Summary method of data ascertainment and differences in questionnaires.

	**1999**	**2002/2003**	**2005**	**2010/2011**	**2017**
Number of participating CHC organizations	39	50	23	17	17
Number of participating CHC centers	170	246	101	na	139
Distribution of questionnaires	To 15 consecutive parents visiting each center	Implemented in “Milk feeding of infants” survey each center To all parents visiting each center	To 15 consecutive parents visiting each center	To 20–40 consecutive parents visiting each center	Flyers with link to online questionnaire 21 CHC centers in low SES areas selected to conduct paper questionnaires directly Link also distributed online
Questionnaires send/received (%)	na/2,845 (na)	4,860/2,913 (60%)	na/1,490 (na)	3,048/2,014 (66%)	9,000^*^/1,289 (14%)
Who filled in questionnaire?	Research assistant with parent(s)	Parent(s) and sent back by post	Research assistant with parent(s)	Parent(s) in waiting room or at home and sent back by post	Parents online Research assistant with parent(s) on paper
Population for analysis	2,534	2,869	1,399	1,956	1,209
Age infants	<10 months	<7 months	<10 months	<12 months	<12 months
Differences questionnaires				
Sleep position	Last 4 weeks	Last night	Last 4 weeks	Last 4 weeks	Last night
Bedding	Last night	Usually at night	Last night	Last night	Last night
Pacifier	In general	In general	na	In general	Last 4 weeks

* *Flyers with link to online questionnaire*.

*na, not available*.

In 1999 and 2005, selected centers were asked to fill out the questionnaires together with parents. In 2010/11, centers were asked to distribute 20–40 questionnaires to parents who could return the questionnaire either directly or by post.

In 2002/03, questions regarding safe sleeping conditions were included in another survey that distributed questionnaires which were returned by post.

For the last survey in 2017, flyers with a link to an online survey were distributed among CHC centers and, the link to the survey was distributed *via* online media. Additionally, 21 centers in low socioeconomic status areas were selected to conduct in person questionnaires with the help of a research assistant directly at the CHC center.

### Data Assessment

The adherence to the Dutch guidelines for the prevention of SIDS and SUDI (8, 19) was assessed with multiple choice surveys. The questions varied only slightly among surveys; differences are indicated in Table 1. The exact questions used in the surveys are summarized in Supplementary Table 1.

### Population

Population characteristics of the five survey populations of infants are described in Table 2. Mothers of infants in the 2017 survey were more often highly educated compared to the other surveys with available data on education level. As this survey was distributed online, instead of directly filled in at the CHC center, a representative distribution of the education levels of the mothers was not guaranteed. Therefore, the data of the 2017 survey were weighted according to the education level distribution of women aged 25–45 in 2017, retrieved from Statistics Netherlands, with the following weighting factors: 0.936 for low education level; 1.292 for medium education level; and 0.867 for high education level.

**Table 2:** Characteristics of the five survey populations.

	**1999**		**2002/2003**		**2005**		**2010/2011**		**2017^$^**	
	***N*** **= 2,534**		***N*** **= 2,787**		***N*** **= 1,399**		***N*** **= 1,955**		***N*** **= 1,192**	
Age (months)^*^	4.6 ± 2.5		2.7 ± 1.5		4.4 ± 2.6		4.8 ± 2.8		5.1 ± 3.4	
0–3 months	1,024	(40.5)	1,926	(67.1)	616	(44.0)	876	(44.9)	482	(40.5)
4–6 months	884	(34.8)	861	(30.0)	425	(30.4)	601	(30.7)	315	(26.4)
7–11 months	626	(24.7)	–	–	358	(25.6)	478	(24.5)	395	(33.1)
**Male gender**	1,297	(51.2)	2,342	(50.3)	747	(53.7)	981	(50.2)	586	(49.2)
**Birthweight ≥2,500**	2,311	(94.4)	2,630	(95.0)	na		1,796	(94.7)	1,143	(95.9)
**Firstborn**	1,203	(47.6)	1,366	(49.0)	628	(45.1)	1,011	(51.7)	658	(55.2)
**Dutch nationality mother**	na		2,623	(94.3)	na		1,727	(90.3)	1,032	(87.0)
**Education level mother**										
Low	na		842	(30.6)	na		372	(19.6)	157	(13.2)
Medium	na		957	(34.7)	na		613	(32.2)	443	(37.2)
High	na		957	(34.7)	na		915	(48.2)	591	(49.6)

*Values are described as mean ± SD, or **N** (%)*.

*Percentages are based on the sample of the population with available data for the concerned characteristic*.

* *The survey in 2002/03 only included infants up to 6 months of age, and the surveys of 1999 and 2005 infants up to 9 months of age*.

$ *Data of the 2017 survey were weighted according to the education level of women aged 25–45 in 2017 in the Netherlands (22). The total population included 1,209 infants, infants with missing data for mother's education level were excluded*.

*na, not available*.

Age categories were defined as: 0–3 months, 4–6 months, 7–12 months of age, corresponding to the current age-specific prevention advice. The population sizes per category are presented in Table 2. As the survey of 2002/03 was only intended for parents of infants under 7 months of age, the last age category of this survey was excluded.

### Data Analysis

Prevalence of risk reducing behavior, according to the current Dutch SUDI prevention advice, was described for all surveys. Because infant care practices, as well as SIDS and SUDI prevention advice, are different per age of the infant, data were presented separately per age category. Age specific messages are included in the tables. For parental smoking, data were presented separately per education level of the mother. Since for the 1999 survey only published results were available, not all categories could be assessed.

A potential difference in prevalence between the 2010/11 and 2017 surveys for the reported risk reducing behaviors was tested with a z-test with α < 0.05.

## Results

The prevalence of risk reducing behaviors by parents of infants up to 12 months of age in the five consecutive surveys are reported in Tables 3–**8**. Significant differences between the last two surveys are indicated in the tables.

**Table 3:** Prevalence of supine sleep position in the five survey populations, including prevalence per age category.

	**1999**		**2002/2003**		**2005**		**2010/2011**		**2017**		**2017 compared with** **2010/2011**	
	***N*** **= 2,534^$^**		***N*** **= 2,780^$^**		***N*** **= 1,250^$^**		***N*** **= 1,709^$^**		***N*** **= 1,181^$^**			
	***n***	**% (95%-CI)**	***n***	**% (95%-CI)**	***n***	**% (95%-CI)**	***n***	**% (95%-CI)**	***n***	**% (95%-CI)**	***z*** **-statistic**	***P*** **-value**
**Supine sleeping**	1,957	90.1 (89.7–92.1)	2,375	85.4 (84.1–86.7)	1,124	89.9 (88.2–91.6)	1,577	92.3 (91.0–93.6)	977	82.7 (80.5–84.9)	−7.92	0.000
0–3 months	709	88.8 (86.7–91.0)	1,607	83.7 (82.0–85.4)	489	88.9 (86.3–91.5)	684	92.7 (90.8–94.6)	415	87.0 (84.0–90.0)	−3.29	0.001
4–6 months	734	92.1 (90.2–94.0)	768	89.3 (87.2–91.4)	357	92.0 (89.3–94.7)	516	93.1 (91.0–95.2)	272	86.3 (82.5–90.1)	−3.26	0.001
7–11 months	514	92.1 (89.9–94.4)	na	na	278	89.1 (85.6–92.6)	377	90.4 (87.6–93.2)	290	74.6 (70.3–78.9)	−6.11	0.000

*The supine position is advised for all infants, at least until the infant can turn prone and back (often around 6 months of age)*.

$ *Percentages are based on the sample of the population with available data for sleeping position. When sleeping position was assessed over the last four weeks, varying sleeping positions were reported for some infants, and these are not included (1999: 381, 2005: 149, 2010/11: 246)*.

*na, not available*.

### Sleep Position

The prevalence of the supine sleeping position fluctuated between 1999 and 2010/11 in the total group as well as the separate age categories (Table 3). In 2017, a significantly lower prevalence was observed in all age categories compared to 2010/11. On average, around 83% of the infants were being placed supine in 2017, whereas this was 92% in 2010/11.

### Sleep Conditions

Not using a duvet increased from 82% in 1999 to 95% in 2017, with no difference between 2010/11 and 2017 (Table 4). The use of a sleep sack fluctuated over time in all age categories. However, its prevalence increased significantly from 2010/11 (48%) to 2017 (55%), in particular among infants aged between 4 and 12 months, leading to the highest use of a sleep sack in the 2017 survey.

**Table 4:** Prevalence of a sleep sack and not using a duvet in the five survey populations, including prevalence per age category.

	**1999**		2002/2003^*^		**2005**		**2010/2011**		**2017**		**2017 compared with** **2010/2011**	
	***N*** **= 2,534^$^**		***N*** **= 2,787^$^**		***N*** **= 1,399^$^**		***N*** **= 1,955^$^**		***N*** **= 1,192^$^**			
	***n***	**% (95%-CI)**	***n***	**% (95%-CI)**	***n***	**% (95%-CI)**	***n***	**% (95%-CI)**	***n***	**% (95%-CI)**	**z-statistic**	***P*** **-value**
**No duvet**	2,079	82.0 (80.5–83.5)	2,592	93.0 (92.1–93.9)	1,307	93.4 (92.1–94.7)	1,873	95.9 (95.0–96.8)	1,136	95.4 (94.2–96.6)	−0.67	0.502
0–3 months	876	85.5 (83.3–87.7)	1,802	93.6 (92.5–94.7)	588	95.5 (93.9–97.1)	850	97.1 (96.0–98.2)	470	97.4 (96.0–98.8)	0.32	0.748
4–6 months	714	80.8 (78.2–83.4)	790	91.8 (90.0–93.6)	389	91.5 (88.8–94.2)	577	96.0 (94.4–97.6)	302	95.7 (93.5–97.9)	−0.22	0.828
7–11 months	489	78.1 (74.9–81.3)	na	na	330	92.2 (89.4–95.0)	446	93.5 (91.3–95.7)	365	92.6 (90.0–95.2)	−0.52	0.602
**Sleep sack**	1,150	45.5 (43.6–47.4)	1,245	44.7 (42.9–46.5)	570	40.7 (38.1–43.3)	933	47.8 (45.6–50.0)	657	55.1 (52.3–57.9)	3.97	0.000
0–3 months	272	26.6 (23.9–29.3)	674	35.0 (32.9–37.1)	140	22.7 (19.4–26.0)	291	33.3 (30.2–36.4)	141	29.2 (25.2–33.2)	−1.55	0.121
4–6 months	474	53.6 (50.3–56.9)	571	66.3 (63.1–69.5)	213	50.1 (45.3–54.9)	346	57.6 (53.6–61.6)	219	69.5 (64.4–74.6)	3.52	0.000
7–11 months	404	64.5 (60.8–68.2)	na	na	217	60.6 (55.5–65.7)	296	62.1 (57.7–66.3)	297	75.3 (69.1–77.9)	4.16	0.000

*A sleep sack can be used from birth on, but is especially advised when the infants starts turning (often around 3–6 months). A duvet is discouraged up to 2 years of age*.

$ *Percentages are based on the sample of the population with available data for bedding*.

* *2002/03 survey assessed usual bedding at night*.

*na, not available*.

The use of a pacifier was lowest in 1999 (Table 5). In 2017, over 50% of infants in all age categories were usually placed to sleep with a pacifier, with the highest prevalence in the lowest age categories. Significantly higher usage in 2017 was observed in infants under 4 months of age compared to 2010/11.

**Table 5:** Prevalence of pacifier use when infants were placed to sleep in the five survey populations, including prevalence per age category.

	**1999**		**2002/2003**		**2005**		**2010/2011**		**2017**		**2017 compared** **with 2010/2011**	
	***N*** **= 2,462^$^**		***N*** **= 2,781^$^**		***N*** **= 1,399^$^**		***N*** **= 1,945^$^**		***N*** **= 1,192^$^**			
	***n***	**% (95%-CI)**	***n***	**% (95%-CI)**	***n***	**% (95%-CI)**	***n***	**% (95%-CI)**	***n***	**% (95%-CI)**	**z-statistic**	***P*** **-value**
**Pacifier**	1,001	40.7 (38.8–42.6)	2,040	73.4 (71.8–75.0)	na	na	1,088	56.0 (53.7–58.1)	699	58.7 (55.9–61.5)	1.48	0.138
0–3 months	331	33.4 (30.5–36.3)	1,499	78.0 (76.1–79.9)	na	na	490	56.2 (52.9–59.5)	303	62.8 (58.5–67.1)	2.36	0.018
4–6 months	364	42.6 (39.3–45.9)	541	62.9 (59.7–66.1)	na	na	342	57.2 (53.2–61.2)	189	60.0 (54.6–65.4)	0.82	0.415
7–11 months	306	49.8 (45.9–53.7)	na	na	na	na	256	54.0 (49.4–58.4)	207	52.4 (47.7–57.5)	−0.47	0.638

*A pacifier is advised when breastfeeding is well established (often around 1 month of age)*.

$ *Percentages are based on the sample of the population with available data for pacifier use*.

*na, not available*.

The prevalence of infants sleeping in a room with their parents and not sharing the bed, increased in all age categories (Table 6). The highest prevalence was found in 2017, when over 30% of the infants shared a room with the parents, and not the bed. For infants 0–3 months old this was over 50%. The difference between 2010/11 and 2017 was significant for all age categories. However, also the prevalence of infants sleeping in their parents' bed increased to 10% in 2017, with over 9% of 0–3 month old infants not sleeping in their own bed or cot during the night (Supplementary Table 2).

**Table 6:** Prevalence of room-sharing during sleep in the five survey populations, including prevalence per age category.

	**1999**		**2002/2003**		**2005**		**2010/2011**		**2017**		**2017 compared** **with 2010/2011**	
	***N*** **= 2,534^$^**		***N*** **= 2,770^$^**		***N*** **= 1,395^$^**		***N*** **= 1,803^$^**		***N*** **= 1,185^$^**			
	***n***	**% (95%-CI)**	***n***	**% (95%-CI)**	***n***	**% (95%-CI)**	***n***	**% (95%-CI)**	***n***	**% (95%-CI)**	**z-statistic**	***P*** **-value**
**Room-sharing not bed**	369	14.6 (13.2–16.0)	640	23.1 (21.5–24.7)	253	18.1 (16.1–20.1)	307	17.0 (15.3–18.7)	362	30.6 (28.0–33.2)	8.72	0.000
0–3 months	222	21.7 (19.2–24.2)	516	27.0 (25.0–29.0)	140	22.8 (19.5–26.1)	211	27.2 (24.1–30.3)	246	51.5 (47.0–56.0)	8.68	0.000
4–6 months	107	12.1 (10.0–14.2)	124	14.5 (12.1–16.9)	84	19.8 (16.0–23.6)	67	11.9 (9.2–14.6)	65	20.6 (16.1–25.1)	3.46	0.001
7–11 months	40	6.4 (4.5–8.3)	na	na	29	8.1 (5.3–10.9)	29	6.2 (4.0–8.4)	52	13.1 (9.8–16.4)	3.46	0.001

*Room-sharing is advised up to 6 months of age, and bed-sharing discouraged until 4 months, or 6 months when parent(s) smoke*.

$ *Percentages are based on the sample of the population with available data for sleeping place*.

*na, not available*.

### Breastfeeding

The prevalence of breastfeeding fluctuated over the surveys and decreased with age as seen in Table 7. In 1999, almost 90% of the infants received any breast milk, either exclusive or in combination with formula milk, the highest prevalence of all surveys. In 2017, infants were more often exclusively breastfed compared to 2010/11 in all age categories.

**Table 7:** Prevalence of feeding type in the five survey populations, including prevalence per age category.

	**1999**		**2002/2003**		**2005**		**2010/2011**		**2017**		**2017 compared** **with 2010/2011**	
	***N*** = 2,534^*^		***N*** = 2,785^*^		***N*** = 1,389^*^		***N*** = 1,797^*^		***N*** = 1,179^*^			
	***n***	**% (95%-CI)**	***n***	**% (95%-CI)**	***n***	**% (95%-CI)**	***n***	**% (95%-CI)**	***n***	**% (95%-CI)**	**z-statistic**	***P*** **-value**
**Exclusive breastfeeding**	501	19.8 (18.2–21.4)	1,001	35.9 (34.1–37.7)	409	29.4 (27.0–31.8)	422	23.5 (21.6–25.4)	370	31.4 (28.8–34.0)	4.77	0.000
0–3 months	334	32.6 (29.7–35.5)	778	40.4 (38.2– 42.6)	267	43.7 (39.8–47.6)	285	35.0 (31.8–38.2)	220	45.8 (41.4–50.2)	3.85	0.000
4–6 months	130	14.7 (12.4–17.0)	223	25.9 (23.0–28.8)	106	25.1 (21.0–29.2)	103	18.6 (15.5–21.7)	78	25.0 (20.2–29.8)	2.22	0.026
7–11 months	37	5.9 (4.1–7.7)	na	na	36	10.1 (7.0–13.2)	34	7.9 (5.5–10.3)	72	18.8 (14.9–22.7)	4.61	0.000
**Mixed breast/formula feeding**	1,751	69.1 (67.3–71.0)	380	13.6 (12.4–14.9)	149	10.7 (9.1–12.3)	191	10.6 (9.2–12.0)	124	10.5 (8.8–12.2)	−0.09	0.931
0–3 months	556	54.3 (51.2–57.4)	249	12.9 (11.4–14.4)	57	9.3 (7.0–11.6)	98	12.0 (9.8–14.2)	52	10.8 (8.0–13.6)	−0.65	0.514
4–6 months	652	73.8 (70.9–76.7)	131	15.2 (12.8–17.6)	51	12.1 (9.0–15.2)	60	10.8 (8.3–13.3)	35	11.2 (7.7–14.7)	0.18	0.856
7–11 months	543	86.7 (84.0–89.4)	na	na	41	11.5 (8.2–14.8)	33	7.7 (5.3–10.1)	37	9.6 (6.7–12.5)	0.97	0.334

*Breastfeeding is advised up to 6 months of age*.

$ *Percentages are based on the sample of the population with available data for type of feeding*.

*na = not available*.

### Smoking

An increase in mothers who abstained from smoking during pregnancy was observed between 2002/03 and 2017, across all education levels (Table 8). Simultaneously, the prevalence of both parents not smoking postpartum increased. Differences in smoking prevalence between education levels of the mothers were observed.

**Table 8:** Prevalence of not smoking by both parents of the infants, and not smoking of mother during pregnancy in the five survey populations, including prevalence per education level of the mother.

	**1999**		**2002/2003**		**2005**		**2010/2011**		**2017**		**2017 compared** **with 2010/2011**	
	***N*** **= 2,507^$^**		***N*** **= 2,787^$^**		***N*** **= 1,399^$^**		***N*** **= 1,955^$^**		***N*** **= 1,192^$^**			
	***n***	**% (95%CI)**	***n***	**% (95%CI)**	***n***	**% (95%CI)**	***n***	**% (95%CI)**	***n***	**% (95%CI)**	**z-statistic**	***P*** **-value**
**Mother did not smoke during pregnancy**	na	na	2,394	85.9 (84.6–87.2)	na	na	na	na	1,144	96.1 (95.0–97.2)	na	na
Low education	na	na	651	78.7 (75.9–81.5)	na	na	na	na	136	86.8 (81.5–92.1)	na	na
Middle education	na	na	833	87.5 (85.4–89.6)	na	na	na	na	424	95.6 (93.7–97.5)	na	na
High education	na	na	888	93.4 (91.8–95.0)	na	na	na	na	584	98.8 (97.9–99.7)	na	na
Both parents do not smoke^*^	1,537	61.3 (59.4–63.2)	1,859	69.3 (67.6–71.0)	887	63.8 (61.3–66.3)	1,338	74.6 (72.7–76.5)	902	77.9 (75.4–80.2)	2.05	0.041
Low education	na	na	474	59.2 (55.9–62.5)	na	na	183	54.3 (49.2–59.4)	95	62.3 (54.4–69.6)	1.65	0.098
Middle education	na	na	623	67.7 (64.7–70.7)	na	na	401	70.5 (66.9–74.1)	297	69.7 (65.4–74.0)	−0.27	0.785
High education	na	na	747	79.8 (77.3–82.3)	na	na	743	85.7 (83.4–88.0)	511	87.9 (85.3–90.5)	1.20	0.229

$ *Percentages are based on the sample of the population with available data for smoking of parent(s). Subcategories are only based on the sample of the population with available data for both education level of the mother and smoking of parents*.

*na, not available*.

## Discussion

The aim of this study was to describe the prevalence of parental behavior recommended in the Dutch infant safe sleeping guidelines and analyze potential changes in prevalence between the latest surveys to identify where current focus is needed. The prevalence of infant sleep position fluctuated over the surveys. Significantly less infants were placed in the supine position in 2017 compared to 2010/11, with more infants sleeping prone or on the side in 2017. More infants were placed to sleep in a sleep sack in 2017 compared to 2010/11. The highest prevalence of room-sharing, but not bed-sharing was found in 2017, however also an increase in bed-sharing was observed. Although parents are advised to room-share for the first 6 months of life, this message seems to be one of the most difficult to adhere to. Type of milk feeding varied per survey, with more infants being breastfed exclusively in all age categories in 2017. An increase in mothers who abstained from smoking during pregnancy, and both parents not smoking in general was observed, although mostly in higher educated parents.

SUDI prevention strategies in the Netherlands has proved to be effective, resulting in a decreasing SUDI incidence and increasing prevalence of most preventive infant care factors over time. However, as visualized in Figure 1, from 2017 on, the SUDI incidence seems to increase again. It is unknown if this reflects yearly fluctuation, or an actual increase. Indications of a lower prevalence of the supine sleeping position and higher prevalence of bed-sharing in 2017, compared to the prior survey, might relate to this potentially increased SUDI incidence. Renewed attention for the current prevention advice is therefore needed.

The incidence of SIDS/SUDI is very low in the Netherlands, which makes it of interest to see if our infant care habits are comparable to other countries. Although the prevalence of infants placed to sleep in the supine position decreased in our study, it is still comparable to Ireland (23) and Australia (24). In Scandinavian countries, only just over 60% of infants were always placed supine (25, 26). Room-sharing is more prevalent in these countries compared to the Netherlands, i.e., 60% in Norway (25), and 54% in Sweden (26). However, also bed-sharing is a very common, perhaps cultural, behavior in Norway and Sweden. While an increased prevalence of bed-sharing in the Netherlands was observed (10% in 2017), the prevalence was 63% in Norway, and in Sweden 43% among infants 3 months of age, and 33% at 6 months (25, 26). In both countries so-called baby nests are a popular sleep surface when bed-sharing. There are concerns about safety of these baby nests (27), but although we advise against them as a sleep surface, they seem to become more popular. Their use needs to be assessed in following surveys. Accurate data about infant care habits are not widely available for countries. It is therefore not possible to hypothesize on their influence on differences in incidence, especially as SIDS seems to be such a multifactorial occurrence.

An infant being placed to sleep in the prone position was associated with a three times higher risk of SUDI compared to the supine position among infants up to 9 months in the Netherlands between 1996 and 2001 (28). Internationally, 2 to 13 times higher risks were found (29). With the lower prevalence of the supine position in this study in 2017, more infants were placed to sleep in the prone (8.9%) and side position (8.4%) compared to earlier surveys. Especially in the lowest age categories, where the SUDI risk is highest, the prevalence of the prone position more than doubled. This can be of great concern in relation to the increased SUDI incidence.

Since the advice in 2004 to room-share with an infant the first 6 months, and not share the sleep surface the first 4 months, infants were more often placed to sleep in the parents' bedroom while not sharing the same bed. These infants have a lower risk of SUDI (30, 31). Nevertheless, the prevalence of so-called room-sharing was only 31% in 2017, and 51% among infants aged under 4 months. The prevalence of bed-sharing was also much lower in 2010/11 compared to the survey in 2005. Nevertheless, in 2017 this prevalence was again at a comparable level to 2005. Bed-sharing of a parent with the infant increases the risk of SUDI, and it is believed that a soft mattress, a duvet, pillows, the risk of overheating and the risk of overlaying can potentially contribute to this increased risk (8, 29). The risk of bed-sharing increases when parents smoke, drink alcohol, use drugs or are extremely tired (30). The Dutch prevention advice focuses on encouraging placing the infant to sleep in its own bed or cot. This prevalence decreased but was still 90% in 2017. However, the higher prevalence of bed-sharing in 2017, comparable to that in 2005, is of great concern. New sleeping practices, such as ‘clip-on beds' or co-sleepers, become more popular and monitoring of their safety seems warranted.

The prevalence of smoking, both during and after pregnancy, greatly decreased over the past twenty years. Nevertheless, low and middle educated groups are lagging behind in this trend (32). Smoking during pregnancy and after birth both need ongoing attention, which in the Netherlands is covered in the national prevention agreement of the Dutch Government (33).

In addition, promoting breastfeeding could contribute to the prevention of SUDI. Breastfeeding could improve in the Netherlands as currently, 69% of the infants receive breastfeeding directly after birth, while at 3 months of age this is only 31%, and this decreases to 19% at 6 months of age (34). Breastfeeding is especially lower among mothers with middle or low education levels. These national numbers have decreased compared to earlier reports (34). In this study however, the prevalence of breastfeeding fluctuated over time, but in 2017 more infants were exclusively breastfed compared to 2010/11 in all age categories. Specific advice targeting middle and low educated parents is needed.

Many governmental institutions promote safe sleeping of infants and thereby contribute to the prevention of SUDI. Preventive youth healthcare is organized differently in different countries, nevertheless, there are many similarities between European countries (35). In the Netherlands, during the first week postpartum, maternity caregivers visit a family on a daily basis, where they provide information and help out with infant care. Furthermore, parents visit the CHC center with their infant about eight times during the first year of life for regular check-ups and to receive information. Despite these valuable channels for communicating the safe sleeping advice, we don't seem to take the current power and influence of the internet into account sufficiently. Many pictures contradict the safe sleeping advice on popular websites, in blogs, in ads, and on social media (20, 21). These pictures influence the behavior of young parents. An intervention providing videos *via* social media was shown to be effective in improving adherence to infant safe sleep practices by changing maternal attitudes and perceived social norms (36, 37). Therefore, it is important to anticipate, and provide more modern, outreaching, picture driven information to reach all young parents, next to all evidence based and well described information on (governmental) institutions' websites. Currently in the Netherlands, a group of parents of SUDI infants closely work together with professionals to take action and tackle inadequate information *via* Instagram.

### Strengths and Limitations

The periodical collection of data on sleep related infant care habits in the Netherlands is unique. With data on the last five surveys over the past 20 years, we were able to monitor prevalences of parental behavior recommended in the national infant safe sleeping guidelines. Parents from different regions in the Netherlands participated in these surveys, ensuring a representative sample of the Dutch population. The surveys were distributed among CHC centers in slightly different, but comparable ways. The last survey used an online questionnaire, where an equal distribution of education levels could not be guaranteed, for which we corrected in this study. Although not validated, most surveys used similar questionnaires, making comparison between them possible. The main difference was in the assessment of the sleeping position of the infant either last night or over the past 4 weeks but, it is not to be expected that this has had major influence on the results. Some bias could have occurred however, due to these differences in questions, and due to the administration difference of online, postal and in person questionnaires. Lastly, parents who filled out the questionnaires were aware of the goal of the survey, namely to monitor safe sleeping of infants. Therefore, it cannot be ruled out that parents may have provided socially desirable answers, and that the actual behavior is less favorable. However, no differences in social desirability are to be expected between the surveys as execution of the studies was similar.

## Conclusion

In a low SUDI incidence country as the Netherlands, attention for prevention advice can weaken for both parents and (professional) caregivers, as well as governmental organizations and (public) health professionals. The possible small increase in SUDI incidence over the last years and a lower prevalence of some risk reducing behaviors in the latest survey, show the importance of renewed attention for the prevention of SUDI. Attention could specifically be aimed at sleeping in the supine position and in an own bed or cot, combined with ongoing attention for the prevention of smoking, especially among lower socioeconomic status groups. Modern, picture driven information *via* social media and the internet could be considered.

## Data Availability Statement

The original contributions presented in the study are included in the article/Supplementary Materials, further inquiries can be directed to the corresponding author.

## Author Contributions

FK, ML'H, MB-B, AE, and EF made substantial contributions to the conception, design, and interpretations of the work. FK performed the analyses of the data. All authors assisted in preparing the article, critically assessed the final version, and agree to be accountable for the accuracy and integrity of the work.

## Funding

This research is funded by the Wageningen University, Department of Human Nutrition and Health and Department of Consumption and Healthy Lifestyles. Furthermore, the research is financially supported by the Dutch Association of Parents of Cot Death Infants (VOWK).

## Conflict of Interest

The authors declare that the research was conducted in the absence of any commercial or financial relationships that could be construed as a potential conflict of interest.

## Publisher's Note

All claims expressed in this article are solely those of the authors and do not necessarily represent those of their affiliated organizations, or those of the publisher, the editors and the reviewers. Any product that may be evaluated in this article, or claim that may be made by its manufacturer, is not guaranteed or endorsed by the publisher.
