# Supplementary material for: Renewed Attention Needed for Prevention of Sudden Unexpected Death in Infancy in the Netherlands
Source: Front Pediatr. 2021 Dec 6;9:757530. doi: 10.3389/fped.2021.757530 (PMC8685403; doi:10.3389/fped.2021.757530)
Supplement: Supplementary file 1 [file Table_1.DOCX]

Supplementary Material

# Supplementary Figures and Tables

|  | 1999 | 2002/2003 | 2005 | 2010/2011 | 2017 |
| --- | --- | --- | --- | --- | --- |
| Sleeping position | ‘In what position was your infant placed to sleep during the night in the last 4 weeks?’ | ‘In what position was your infant placed to sleep last night?’ | ‘In what position was your infant placed to sleep during the night in the last 4 weeks?’ | ‘In what position was your infant placed to sleep during the night in the last 4 weeks?’ | ‘In what position was your infant placed to sleep last night?’ |
| Bedding (duvet and sleep sack) | ‘What was your child covered with last night?’ | ‘Indicate what your child is usually covered with at night.’ | ‘What was your child covered with last night?’ | ‘What was your child covered with last night?’ | ‘What was your child covered with last night?’ |
| Pacifier | ‘Is your infant placed to sleep with a pacifier?’ | ‘Is your infant placed to sleep with a pacifier?’ | NA | ‘Is your infant placed to sleep with a pacifier?’ | ‘Was your infant placed to sleep with a pacifier in the last 4 weeks?’ |
| Sleeping place | ‘Where is your infant usually sleeping during the night?’ | ‘Where is your infant usually sleeping during the night?’ | ‘Is your infant usually sleeping in the parents’ bedroom during the night?’  ‘Does the infant sleep with the parents in bed during the night?’ | ‘Where is your infant usually sleeping during the night?’ | ‘Where is your infant usually sleeping during the night?’  ‘Where does the infant sleep when sleeping in the parents’ bedroom?’ |
| Smoking | ‘Does the mother/father smoke?’ | ‘Does the mother/father smoke?’  ‘Did mother smoke during pregnancy?’ | ‘Does the mother/father smoke?’ | ‘Does the mother/father smoke?’ | ‘Does the mother/father smoke?’  ‘Did mother smoke during pregnancy?’ |
| Feeding type | What type of milk feeding is your infant receiving at the moment?’ | What type of milk feeding is your infant receiving at the moment?’ | What type of milk feeding is your infant receiving at the moment?’ | What type of milk feeding is your infant receiving at the moment?’ | What type of milk feeding is your infant receiving at the moment?’ |

**Supplementary Table 1.** Questions translated from questionnaires used to assess adherence to Dutch guidelines for the prevention of SIDS and SUDI.

**Supplementary Table 2.** Prevalence of positions infants were placed in to sleep in the five survey populations, including prevalence per age category. The supine position is advised for all infants, at least until the infant can turn prone and back (often around 6 months of age).

|  | 1999 | | 2002/2003 | | 2005 | | 2010/2011 | | 2017 | |
| --- | --- | --- | --- | --- | --- | --- | --- | --- | --- | --- |
|  | n=2153^$^ | | n=2780^$^ | | n=1250^$^ | | n=1709^$^ | | n=1181^$^ | |
|  | freq | % (95%-CI) | freq | % (95%-CI) | freq | % (95%-CI) | freq | % (95%-CI) | freq | % (95%-CI) |
| Prone sleeping | 74 | 3.4 (2.7-4.2) | 78 | 2.8 (2.2-3.4) | 42 | 3.4 (2.4-4.4) | 52 | 3.0 (2.2-3.8) | 105 | 8.9 (7.3-10.5) |
| 0-3 months | 10 | 1.3 (0.5-2.0) | 50 | 2.6 (1.9-3.3) | 12 | 2.2 (1.0-3.4) | 11 | 1.5 (0.6-2.4) | 23 | 4.8 (2.9-6.7) |
| 4-6 months | 39 | 4.9 (3.4-6.4) | 28 | 3.3 (2.1-4.5) | 15 | 3.9 (2.0-5.8) | 18 | 3.2 (1.7-4.7) | 21 | 6.7 (3.9-9.5) |
| 7-11 months | 25 | 4.5 (2.8-6.2) | na | na | 15 | 4.8 (2.4-7.2) | 23 | 5.5 (3.3-7.7) | 61 | 15.6 (12.0-19.2) |
| Side sleeping | 122 | 5.7 (4.7-6.6) | 327 | 11.8 (10.6-13.0) | 84 | 6.7 (5.3-8.1) | 80 | 4.7 (3.7-5.7) | 99 | 8.4 (6.8-10.0) |
| 0-3 months | 79 | 9.9 (7.8-12.0) | 263 | 13.7 (12.2-15.2) | 49 | 8.9 (6.5-11.3) | 43 | 5.8 (4.1-7.5) | 39 | 8.2 (5.8-10.6) |
| 4-6 months | 24 | 3.0 (1.8-4.2) | 64 | 7.4 (5.7-9.2) | 16 | 4.1 (2.1-6.1) | 20 | 3.6 (2.0-5.2) | 22 | 7.1 (4.3-9.9) |
| 7-11 months | 19 | 3.4 (1.9-4.9) | na | na | 19 | 6.1 (3.4-8.8) | 17 | 4.1 (2.2-6.0) | 38 | 9.8 (6.9-12.7) |
| Supine sleeping | 1957 | 90.1 (89.7-92.1) | 2375 | 85.4 (84.1-86.7) | 1124 | 89.9 (88.2-91.6) | 1577 | 92.3 (91.0-93.6) | 977 | 82.7 (80.5-84.9) |
| 0-3 months | 709 | 88.8 (86.7-91.0) | 1607 | 83.7 (82.0-85.4) | 489 | 88.9 (86.3-91.5) | 684 | 92.7 (90.8-94.6) | 415 | 87.0 (84.0-90.0) |
| 4-6 months | 734 | 92.1 (90.2-94.0) | 768 | 89.3 (87.2-91.4) | 357 | 92.0 (89.3-94.7) | 516 | 93.1 (91.0-95.2) | 272 | 86.3 (82.5-90.1) |
| 7-11 months | 514 | 92.1 (89.9-94.4) | na | na | 278 | 89.1 (85.6-92.6) | 377 | 90.4 (87.6-93.2) | 290 | 74.6 (70.3-78.9) |

^$^Percentages are based on the sample of the population with available data for sleeping position. When sleeping position was assessed over the last four weeks, variable sleeping position was observed for some infants, and these are not included (1999: 381, 2005: 149, 2010/2011: 246).

**Supplementary Table 3.** Prevalence of duvet and sleep sack use in the five survey populations, including prevalence per age category. A sleep sack can be used from birth on, but is especially advised when the infants starts turning (often around 3-6 months). A duvet is discouraged up to 2 years of age.

|  | 1999 | | 2002/2003 | | 2005 | | 2010/2011 | | 2017 | |
| --- | --- | --- | --- | --- | --- | --- | --- | --- | --- | --- |
|  | n=2534^$^ | | n=2787^$^ | | n=1399^$^ | | n=1953^$^ | | n=1192^$^ | |
|  | freq | % (95%-CI) | freq | % (95%-CI) | freq | % (95%-CI) | freq | % (95%-CI) | freq | % (95%-CI) |
| Duvet | 455 | 18.0 (16.5-19.5) | 195 | 7.0 (6.1-7.9) | 92 | 6.6 (5.3-7.9) | 80 | 4.1 (3.2-5.0) | 55 | 4.6 (3.4-5.8) |
| 0-3 months | 148 | 14.5 (12.3-16.7) | 124 | 6.4 (5.3-7.5) | 28 | 4.5 (2.9-6.1) | 25 | 2.9 (1.8-4.0) | 12 | 2.6 (1.2-4.0) |
| 4-6 months | 170 | 19.2 (16.6-21.8) | 71 | 8.2 (6.4-10.0) | 36 | 8.5 (5.8-11.2) | 24 | 4.0 (2.4-5.6) | 14 | 4.3 (2.1-6.5) |
| 7-11 months | 137 | 21.9 (18.7-25.1) | na | na | 28 | 7.8 (5.0-10.6) | 31 | 6.5 (4.3-8.7) | 29 | 7.4 (4.8-10.0) |
| No duvet | 2079 | 82.0 (80.5-83.5) | 2592 | 93.0 (92.1-93.9) | 1307 | 93.4 (92.1-94.7) | 1873 | 95.9 (95.0-96.8) | 1136 | 95.4 (94.2-96.6) |
| 0-3 months | 876 | 85.5 (83.3-87.7) | 1802 | 93.6 (92.5-94.7) | 588 | 95.5 (93.9-97.1) | 850 | 97.1 (96.0-98.2) | 470 | 97.4 (96.0-98.8) |
| 4-6 months | 714 | 80.8 (78.2-83.4) | 790 | 91.8 (90.0-93.6) | 389 | 91.5 (88.8-94.2) | 577 | 96.0 (94.4-97.6) | 302 | 95.7 (93.5-97.9) |
| 7-11 months | 489 | 78.1 (74.9-81.3) | na | na | 330 | 92.2 (89.4-95.0) | 446 | 93.5 (91.3-95.7) | 365 | 92.6 (90.0-95.2) |
| Sleep sack | 1150 | 45.5 (43.6-47.4) | 1245 | 44.7 (42.9-46.5) | 570 | 40.7 (38.1-43.3) | 933 | 47.8 (45.6-50.0) | 657 | 55.1 (52.3-57.9) |
| 0-3 months | 272 | 26.6 (23.9-29.3) | 674 | 35.0 (32.9-37.1) | 140 | 22.7 (19.4-26.0) | 291 | 33.3 (30.2-36.4) | 141 | 29.2 (25.2-33.2) |
| 4-6 months | 474 | 53.6 (50.3-56.9) | 571 | 66.3 (63.1-69.5) | 213 | 50.1 (45.3-54.9) | 346 | 57.6 (53.6-61.6) | 219 | 69.5 (64.4-74.6) |
| 7-11 months | 404 | 64.5 (60.8-68.2) | na | na | 217 | 60.6 (55.5-65.7) | 296 | 62.1 (57.7-66.3) | 297 | 75.3 (69.1-77.9) |
| No sleep sack | 1384 | 54.6 (52.7-56.5) | 1542 | 55.3 (53.5-57.1) | 829 | 59.3 (56.7-61.9) | 1020 | 52.2 (50.0-54.4) | 535 | 44.9 (42.1-47.7) |
| 0-3 months | 752 | 73.4 (70.7-76.1) | 1252 | 65.0 (62.9-67.1) | 476 | 77.3 (74.0-80.6) | 584 | 66.7 (63.6-69.8) | 341 | 70.8 (66.7-74.9) |
| 4-6 months | 410 | 46.4 (43.1-49.7) | 290 | 33.7 (30.5-36.9) | 212 | 49.9 (45.1-54.7) | 255 | 42.4 (38.4-46.4) | 96 | 30.5 (25.4-35.6) |
| 7-11 months | 222 | 35.3 (31.6-39.0) | na | na | 141 | 39.4 (34.3-44.5) | 181 | 37.9 (33.5-42.3) | 97 | 24.7 (20.4-29.0) |

^$^Percentages are based on the sample of the population with available data for bedding.

**Supplementary Table 4.** Prevalence of pacifier use when infants were placed to sleep in the five survey populations, including prevalence per age category. A pacifier is advised when breastfeeding is well established (often around 1 month of age).

|  | 1999 | | 2002/2003 | | 2005 | | 2010/2011 | | 2017 | |
| --- | --- | --- | --- | --- | --- | --- | --- | --- | --- | --- |
|  | n=2462^$^ | | n=2781^$^ | | n=1399^$^ | | n=1944^$^ | | n=1192^$^ | |
|  | freq | % (95%-CI) | freq | % (95%-CI) | freq | % (95%-CI) | freq | % (95%-CI) | freq | % (95%-CI) |
| Pacifier | 1001 | 40.7 (38.8-42.6) | 2040 | 73.4 (71.8-75.0) | na | na | 1088 | 56.0 (53.7-58.1) | 699 | 58.7 (55.9-61.5) |
| 0-3 months | 331 | 33.4 (30.5-36.3) | 1499 | 78.0 (76.1-79.9) | na | na | 490 | 56.2 (52.9-59.5) | 303 | 62.8 (58.5-67.1) |
| 4-6 months | 364 | 42.6 (39.3-45.9) | 541 | 62.9 (59.7-66.1) | na | na | 342 | 57.2 (53.2-61.2) | 189 | 60.0 (54.6-65.4) |
| 7-11 months | 306 | 49.8 (45.9-53.7) | na | na | na | na | 256 | 54.0 (49.4-58.4) | 207 | 52.6 (47.7-57.5) |
| No pacifier | 1461 | 59.3 (57.4-61.3) | 741 | 26.6 (25.0-28.2) | na | na | 856 | 44.0 (41.8-46.2) | 493 | 41.4 (38.6-44.2) |
| 0-3 months | 661 | 66.6 (63.7-69.6) | 422 | 22.0 (20.1-23.9) | na | na | 382 | 43.8 (40.5-47.1) | 179 | 37.1 (32.8-41.5) |
| 4-6 months | 491 | 57.4 (54.1-60.7) | 319 | 37.1 (33.9-40.3) | na | na | 256 | 42.8 (38.8-46.8 | 126 | 40.0 (34.6-45.4) |
| 7-11 months | 309 | 50.2 (46.3-54.2) | na | na | na | na | 218 | 46.0 (41.5-50.5) | 188 | 47.6 (42.7-52.5) |

^$^Percentages are based on the sample of the population with available data for pacifier use.

**Supplementary Table 5.** Prevalence of room- and bed-sharing during sleep in the five survey populations, including prevalence per age category. Room-sharing is advised up to 6 months of age, and bed-sharing discouraged until 4 months, or 6 months when parent(s) smoke.

|  | 1999 | | 2002/2003 | | 2005 | | 2010/2011 | | 2017 | |
| --- | --- | --- | --- | --- | --- | --- | --- | --- | --- | --- |
|  | n=2534^$*^ | | n=2770^$^ | | n=1395^$^ | | n=1803^$^ | | n=1185^$^ | |
|  | freq | % (95%-CI) | freq | % (95%-CI) | freq | % (95%-CI) | freq | % (95%-CI) | freq | % (95%-CI) |
| Room-sharing not bed | 369 | 14.6 (13.2-16.0) | 640 | 23.1 (21.5-24.7) | 253 | 18.1 (16.1-20.1) | 307 | 17.0 (15.3-18.7) | 362 | 30.6 (28.0-33.2) |
| 0-3 months | 222 | 21.7 (19.2-24.2) | 516 | 27.0 (25.0-29.0) | 140 | 22.8 (19.5-26.1) | 211 | 27.2 (24.1-30.3) | 246 | 51.5 (47.0-56.0) |
| 4-6 months | 107 | 12.1 (10.0-14.2) | 124 | 14.5 (12.1-16.9) | 84 | 19.8 (16.0-23.6) | 67 | 11.9 (9.2-14.6) | 65 | 20.6 (16.1-25.1) |
| 7-11 months | 40 | 6.4 (4.5-8.3) | na | na | 29 | 8.1 (5.3-10.9) | 29 | 6.2 (4.0-8.4) | 52 | 13.1 (9.8-16.4) |
| Room- and bed-sharing | 225 | 8.9 (7.8-10.0) | 136 | 4.9 (4.1-5.7) | 141 | 10.1 (8.5-11.7) | 101 | 5.6 (4.5-6.7) | 118 | 10.0 (8.3-11.7) |
| 0-3 months | 111 | 10.8 (8.9-12.7) | 103 | 5.4 (4.4-6.4) | 83 | 13.5 (10.8-16.2) | 52 | 6.7 (4.9-8.5) | 44 | 9.1 (6.5-11.7) |
| 4-6 months | 61 | 6.9 (5.2-8.6) | 33 | 3.9 (2.6-5.2) | 37 | 8.7 (6.0-11.4) | 26 | 4.6 (2.9-6.3) | 24 | 7.7 (4.8-10.6) |
| 7-11 months | 53 | 8.5 (6.3-10.7) | na | na | 21 | 5.9 (3.5-8.3) | 23 | 4.9 (2.9-6.9) | 51 | 12.9 (9.6-16.2) |
| No room-sharing | 2013 | 79.4 (77.8-81.0) | 1994 | 72.0 (70.3-73.7) | 1001 | 71.8 (69.4-74.2) | 1395 | 77.4 (75.5-79.3) | 704 | 59.4 (56.6-62.2) |
| 0-3 months | 731 | 71.4 (68.6-74.2) | 1294 | 67.6 (65.5-69.7) | 391 | 63.7 (59.9-675) | 513 | 66.1 (62.8-69.4) | 188 | 39.4 (35.0-43.8) |
| 4-6 months | 737 | 83.4 (80.9-85.9) | 700 | 81.7 (79.1-84.3) | 304 | 71.5 (67.2-75.8) | 469 | 83.5 (80.4-86.6) | 225 | 71.7 (66.7-76.7) |
| 7-11 months | 545 | 87.1 (84.5-89.7) | na | na | 306 | 86.0 (82.4-89.6) | 413 | 88.8 (85.9-91.7) | 291 | 74.0 (69.7-78.3) |

^$^Percentages are based on the sample of the population with available data for sleeping place.
*Percentages in the 1999 survey exceeded 100% as more answers for sleeping position in the last night were possible.

**Supplementary Table 6.** Prevalence of feeding type in the five survey populations, including prevalence per age category. Breastfeeding is advised up to 6 months of age.

|  | 1999 | | 2002/2003 | | 2005 | | 2010/2011 | | 2017 | |
| --- | --- | --- | --- | --- | --- | --- | --- | --- | --- | --- |
|  | n=2534^$^ | | n=2785^$^ | | n=1389^$^ | | n=1797^$^ | | n=1179^$^ | |
|  | freq | % | freq | % (95%-CI) | freq | % (95%-CI) | freq | % (95%-CI) | freq | % (95%-CI) |
| Exclusive breastfeeding | 501 | 19.8 (18.2-21.4) | 1001 | 35.9 (34.1-37.7) | 409 | 29.4 (27.0-31.8) | 422 | 23.5 (21.6-25.4) | 370 | 31.4 (28.8-34.0) |
| 0-3 months | 334 | 32.6 (29.7-35.5) | 778 | 40.4 (38.2- 42.6) | 267 | 43.7 (39.8-47.6) | 285 | 35.0 (31.8-38.2) | 220 | 45.8 (41.4-50.2) |
| 4-6 months | 130 | 14.7 (12.4-17.0) | 223 | 25.9 (23.0-28.8) | 106 | 25.1 (21.0-29.2) | 103 | 18.6 (15.5-21.7) | 78 | 25.0 (20.2-29.8) |
| 7-11 months | 37 | 5.9 (4.1-7.7) | na | na | 36 | 10.1 (7.0-13.2) | 34 | 7.9 (5.5-10.3) | 72 | 18.8 (14.9-22.7) |
| Exclusive formula feeding | 282 | 11.1 (9.9-12.3) | 1404 | 50.4 (48.5-52.2) | 831 | 59.8 (57.2-62.4) | 1184 | 65.9 (63.8-68.0) | 684 | 58.1 (55.3-60.9) |
| 0-3 months | 134 | 13.1 (11.0-15.2) | 897 | 46.6 (44.3-48.8) | 287 | 47.0 (43.1-50.9) | 431 | 52.9 (49.6-56.2) | 209 | 43.4 (39.0-47.8) |
| 4-6 months | 102 | 11.5 (9.4-13.6) | 507 | 58.9 (55.6-62.2) | 266 | 62.9 (58.3-67.5) | 391 | 70.6 (67.0-74.2) | 199 | 63.9 (58.6-69.2) |
| 7-11 months | 46 | 7.4 (5.3-9.5) | na | na | 278 | 78.3 (74.0-82.6) | 362 | 84.4 (81.2-87.6) | 276 | 71.6 (67.2-76.0) |
| Mixed breast/formula feeding | 1751 | 69.1 (67.3-71.0) | 380 | 13.6 (12.4-14.9) | 149 | 10.7 (9.1-12.3) | 191 | 10.6 (9.2-12.0) | 124 | 10.5 (8.8-12.2) |
| 0-3 months | 556 | 54.3 (51.2-57.4) | 249 | 12.9 (11.4-14.4) | 57 | 9.3 (7.0-11.6) | 98 | 12.0 (9.8-14.2) | 52 | 10.8 (8.0-13.6) |
| 4-6 months | 652 | 73.8 (70.9-76.7) | 131 | 15.2 (12.8-17.6) | 51 | 12.1 (9.0-15.2) | 60 | 10.8 (8.3-13.3) | 35 | 11.2 (7.7-14.7) |
| 7-11 months | 543 | 86.7 (84.0-89.4) | na | na | 41 | 11.5 (8.2-14.8) | 33 | 7.7 (5.3-10.1) | 37 | 9.6 (6.7-12.5) |

^$^Percentages are based on the sample of the population with available data for type of feeding.

**Supplementary Table 7.** Prevalence of parental smoking, and smoking of mother during pregnancy in the five survey populations, including prevalence per education level of the mother.

|  | 1999 | | 2002/2003 | | 2005 | | 2010/2011 | | 2017 | |
| --- | --- | --- | --- | --- | --- | --- | --- | --- | --- | --- |
|  | n=2507^$^ | | n=2787^$^ | | n=1399^$^ | | n=1955^$^ | | n=1192^$^ | |
|  | freq | % (95%CI) | freq | % (95%CI) | freq | % (95%CI) | freq | % (95%CI) | freq | % (95%CI) |
| Mother smoked during pregnancy | na | na | 365 | 14.1 (12.8-15.4) | na | na | na | na | 47 | 3.9 (2.8-5.0) |
| Low education | na | na | 176 | 21.3 (18.5-24.1) | na | na | na | na | 21 | 13.2 (7.9-18.5) |
| Middle education | na | na | 119 | 12.5 (10.4-14.6) | na | na | na | na | 19 | 4.4 (2.5-6.3) |
| High education | na | na | 63 | 6.6 (5.0-8.2) | na | na | na | na | 7 | 1.2 (0.3-2.1) |
| Mother did not smoke during pregnancy | na | na | 2394 | 85.9 (84.6-87.2) | na | na | na | na | 1144 | 96.1 (95.0-97.2) |
| Low education | na | na | 651 | 78.7 (75.9-81.5) | na | na | na | na | 136 | 86.8 (81.5-92.1) |
| Middle education | na | na | 833 | 87.5 (85.4-89.6) | na | na | na | na | 424 | 95.6 (93.7-97.5) |
| High education | na | na | 888 | 93.4 (91.8-95.0) | na | na | na | na | 584 | 98.8 (97.9-99.7) |
| One of parents smokes^*^ | 970 | 38.7 (36.8-40.6) | 825 | 30.7 (29.0-32.4) | 503 | 36.2 (33.7-38.7) | 456 | 25.4 (23.5-27.5) | 257 | 22.1 (19.7-24.5) |
| Low education | na | na | 327 | 40.8 (37.5-44.1) | na | na | 154 | 45.7 (40.8-51.0) | 57 | 37.7 (30.0-45.4) |
| Middle education | na | na | 297 | 32.3 (29.3-35.3) | na | na | 168 | 29.5 (25.9-33.1) | 129 | 30.3 (25.9-34.7) |
| High education | na | na | 189 | 20.2 (17.7-22.7) | na | na | 124 | 14.3 (12.0-16.6) | 70 | 12.1 (9.4-14.8) |
| Both parents do not smoke^*^ | 1537 | 61.3 (59.4-63.2) | 1859 | 69.3 (67.6-71.0) | 887 | 63.8 (61.3-66.3) | 1338 | 74.6 (72.7-76.5) | 902 | 77.9 (75.4-80.2) |
| Low education | na | na | 474 | 59.2 (55.9-62.5) | na | na | 183 | 54.3 (49.2-59.4) | 95 | 62.3 (54.4-69.6) |
| Middle education | na | na | 623 | 67.7 (64.7-70.7) | na | na | 401 | 70.5 (66.9-74.1) | 297 | 69.7 (65.4-74.0) |
| High education | na | na | 747 | 79.8 (77.3-82.3) | na | na | 743 | 85.7 (83.4-88.0) | 511 | 87.9 (85.3-90.5) |

$Percentages are based on the sample of the population with available data for smoking of parent(s). Subcategories are only based on the sample of the population with available data for both education level of the mother and smoking of parents.
